# Age Modulates the Effects of Mental Fatigue on Typewriting

**DOI:** 10.3389/fpsyg.2018.01113

**Published:** 2018-07-05

**Authors:** Marlon de Jong, Jacob Jolij, André Pimenta, Monicque M. Lorist

**Affiliations:** ^1^Department of Experimental Psychology, Faculty of Behavioral and Social Sciences, University of Groningen, Groningen, Netherlands; ^2^ALGORITMI/Department of Informatics, Universidade do Minho, Braga, Portugal; ^3^Neuroimaging Center Groningen, University Medical Center Groningen, University of Groningen, Groningen, Netherlands

**Keywords:** mental fatigue, aging, typewriting, performance, ecologically sensitive, EEG, P3

## Abstract

In the present study, we examined whether age influences the effects of mental fatigue on task performance, and if we could validate the use of measures based on typing behavior as an index of the effects of mental fatigue on different aspects of cognition. Young (*N* = 24, 18–30 years) and middle-aged (*N* = 24, 50–67 years) participants performed a typewriting task and a mouse targeting task for 120 min. At the beginning and at the end of the experiment the level of subjective fatigue was assessed. During task performance measures based on typing behavior and EEG were recorded. Results showed that subjective fatigue increased over the experiment in both the young and the middle-aged group. Typing speed decreased with time-on-task (ToT) in both age groups, reflected in larger general interkey intervals and in an increase in typing time. In addition, typing accuracy decreased with ToT in the young group, however, not in the middle-aged group, reflected by an increase in typing errors. Moreover, the young group used the backspace key more often with ToT due to delayed error-correction, reflected in larger backspace sequences, resulting in larger interkey intervals and increased typing time. This effect was absent in the middle-aged group. In the young group, the P3 brain potential amplitude decreased over the experiment, which was related to an increase in typing time, longer general interkey intervals, and an increase in typing errors, suggesting that decreased task engagement was related to less efficient typewriting, at least in the young group. These results indicate that measures based on typing behavior could give information about the process of mental fatigue, and in addition suggest that age influences the effect of mental fatigue on typewriting. More specifically, younger adults more often adopt a strategy that emphasizes speed, while middle-aged adults act more error-aversive than younger adults.

## Introduction

Mental fatigue refers to a complex, and multifaceted state that may occur during or after prolonged task performance. Although the effects vary within and between people, mental fatigue is often manifested in aversive feelings against the current task and deteriorations in task performance. More specifically, with time-on-task (ToT) people often perform more slowly, make more mistakes, and are less able to correct for these mistakes (Lorist et al., 2005; Boksem et al., 2006).

Since the early 1900s, the effects of mental fatigue and its implications for daily life, and working life in particular, have been addressed by numerous researchers (e.g., Thorndike, 1900; Muscio, 1921; Bartley and Chute, 1949; Ackerman, 2011; Hockey, 2013). In order to explain the effects of mental fatigue and the cognitive processes involved, several theoretical models have been developed. Bartlett (1941), for example, argued that the effects of mental fatigue are related to a decline in cognitive control, resulting in a decreased ability to coordinate and accurately time complex activities, which is accompanied by a growing irritability and awareness of physical discomfort. Kanfer and Ackerman (1989) put forward that both a decrease in cognitive resources and a decline in the motivation to allocate the residual resources to the current task play a role in explaining the effects of mental fatigue. More recently, mental fatigue has been argued to develop as a result of a cost-benefit evaluation of effort and might serve as a warning system for a decrease in performance efficiency; if the costs of performing a cognitive task come to exceed the benefits of finishing the task, subjective experience of mental fatigue (e.g., aversion against task performance, low vigilance) emerges and performance deteriorates (i.e., increase reaction times and decrease accuracy) (Boksem and Tops, 2008). This subjective warning system is not optimal, however. Previous research has shown that people are not able to accurately decide whether they are still capable of effectively performing a task. Comparing performance and subjective measurements, Zhang et al. (2011), for example, showed that performance already declines before people actually become aware of being fatigued. This has real-world consequences: mental fatigue not only affects productivity (Ricci et al., 2007), but it is also one of the most frequent causes of accidents in a working environment (Baker et al., 1994; McCormick et al., 2012).

The effects of mental fatigue can be influenced by internal (e.g., motivation; Boksem et al., 2006) and external (e.g., caffeine; Lorist et al., 1994) factors. Age is an important example of one such factor that might influence the manner in which mental fatigue is expressed. Given that over the past century the average age of the working population has increased, it is important to gain a better understanding of the effects of mental fatigue in older adults in working environments. Although relatively little is known about the interaction between age and mental fatigue on performance, in general, older adults are slower to respond (Verhaeghen and Salthouse, 1997), and act more error-aversive than younger adults, while younger adults are faster and make more mistakes than older adults (Starns and Ratcliff, 2010). The main question in the present study was whether and how the effects of mental fatigue are modulated by age. Because of the differences in overall processing speed and error-aversion, we hypothesized that the effects of mental fatigue are expressed differently in younger and older adults, as well.

Research on mental fatigue in ecologically valid settings has clearly established the implications of mental fatigue in the working environment (e.g., Baker et al., 1994; Ricci et al., 2007; McCormick et al., 2012; Hockey, 2013). One way to monitor performance during a typical working day for office workers, who are prone to mental fatigue, is by using computer input devices (e.g., keyboard and mouse). For example, Pimenta et al. (2014) used keyboard-based performance measures of speed and accuracy to monitor changes in typewritten performance during working days. Based on effects of time-of-day, Pimenta et al. (2014) argued that these keyboard-based performance measures could be used to monitor mental fatigue. Additionally, Kalfaoğlu and Stafford (2014) examined the relation between typing errors and speed. They investigated how typists behaved before, during, and after correct typewriting compared to incorrect typewriting, and found that typists often slow down during and after mistakes, while speed is constant during correct typewriting. Moreover, these mistakes can be predicted by increased variability in typing speed before making mistakes. These results indicate that typing characteristics are susceptible to variability in performance, and may thus be used as a proxy for studying and monitoring mental fatigue.

Although monitoring typewriting behavior might increase our knowledge about the consequences of mental fatigue in the working environment, it should be noted that behavioral measures only describe the consequences of mental fatigue on behavior. That is, behavior is the final result of many different cognitive processes that can be influenced by internal (e.g., mental fatigue) and external factors (e.g., task demands). Measures of behavior can provide some understanding about these cognitive operations, however, they do not give information about these processes directly and therefore their relation with these underlying cognitive processes remain elusive. Measuring brain activity using electro-encephalography (EEG) can offer direct insight in the cognitive processes underlying task performance. Event-related potentials (ERPs) can be extracted from the EEG signal and describe the timing and organization of cognitive processes before, during, or after external (e.g., stimulus presentation) or internal (e.g., making a mistake) events. The P1 (i.e., a positive wave between 100 and 160 ms after stimulus presentation) and N1 (i.e., a negative wave between 160 and 210 ms after stimulus presentation) ERP components, for example, are associated with early processing of visual information (Luck, 2005, p. 36–37). The N400, a negativity wave in the 250–500 ms interval after stimulus presentation, was found to be inversely correlated with the semantic fit of a presented word in the context of a sentence (Kutas and Hillyard, 1984; Hoeks et al., 2004). The P3, a positive wave between 300 and 500 ms after stimulus presentation, has been associated with attention and memory operations (Polich, 2007). Although the exact mechanisms underlying the P3 are unclear and topic of debate, it is known that mental fatigue and decreases in performance, are associated with decreased P3 amplitudes (Lorist and Jolij, 2012; Wascher and Getzmann, 2014; Hopstaken et al., 2015). The second aim of this study was to elucidate the relation between behavioral indices of typing behavior and underlying cognitive processes. To validate if indices of typing behavior could monitor the effects of mental fatigue on specific cognitive operations, we recorded brain activity in addition to behavioral measures.

In sum, in the present study we examined whether age influences the effects of mental fatigue on task performance. During the experiment, participants performed a typewriting task [i.e., an adapted version of the task of Hoeks et al. (2004)] and a mouse targeting task for 2 h. ToT was used to induce mental fatigue. In line with previous aging research (Hertzog et al., 1993; Verhaeghen and Salthouse, 1997; Starns and Ratcliff, 2010), we hypothesized that speed would decline more with ToT in the middle-aged group than in the young group, and accuracy would decline more in the young group than in the middle-aged group. Based on different measures derived from typing behavior we examined different aspects of human performance. Besides measures of typing speed (typing time, general interkey interval, and stimulus–key interval) and typing accuracy (incorrect words, corrected words, backspace use, and backspace sequence length), we investigated the letter–letter interval, an index for speed during correct typewriting, the letter–backspace interval, which is an index for the initiation of error-correction, and the backspace–backspace interval, which is an index of speed during error-correction. With regard to the relation between effects of mental fatigue on different measures of typing behavior and underlying cognitive processes, we expected that top-down cognitive control (e.g., attentional and working memory processes) would be most prone to the effects of mental fatigue during typewriting. Therefore, we hypothesized a decrease of the P3 amplitude over the experiment and an increase in P3 latency with ToT. Furthermore, we expected that changes in the P3 component would be related to fatigue-related changes in typewritten performance.

## Materials and Methods

### Participants

Twenty-four healthy young adults (8 males), ranging in age from 18 to 30 (*M* = 22.4, *SD* = 3.4), and 24 healthy middle-aged adults (11 males), ranging in age from 50 to 67 (*M* = 57.8, *SD* = 6.0) gave their written informed consent to participate in this experiment that was approved by the local Ethics Committee. All participants were Dutch, right-handed, had normal or corrected-to-normal visual acuity. Both the young and the middle-aged participants worked at least 1 h every day on a computer (Young: *M* = 4.0, *SD* = 2.2; Middle-aged: *M* = 4.6, *SD* = 2.9). Sixteen young and 13 middle-aged participants were capable of typing according to the 10-finger system without looking at the keyboard. The participants were not dyslectic, did not use prescription medication, and did not work night shifts. The young adults were students who received either course credits or 20 euro in exchange for their participation; the middle-aged adults were recruited via partner companies of the SPRINT@Work project, local media outlets, social media, and personal contact, and received a lunch or 20 euro in exchange for their participation. Data from two participants (one participant in each age group) was excluded from the analysis due to excessive noise in the EEG data (>30% of the epochs).

### Apparatus and Materials

The experiment was conducted in a sound-attenuated room. The experimental setup consisted of an office chair placed behind an adjustable desk, a windows computer with a QWERTY keyboard, screen support, and an Iiyama prolite G2773HS monitor, model PL2773H. The desk-top lighting was fixed at 800 lux, set according to the Dutch norms for lighting in an office environment. The experimental task was written in PsychToolbox version 3.0 (Brainard, 1996; Pelli, 1997; Kleiner et al., 2007), a toolbox for Matlab R14b (Matlab, 2014). Performance data were acquired using PsychToolbox and a keylogging utility (CAMCof, version 7/10/14; Pimenta et al., 2014). EEG activity was recorded using 20 Sn electrodes on an electrode cap (ElectroCap International). EEG analysis was performed using Matlab R14b in combination with EEGlab (Delorme and Makeig, 2004). Statistical analysis was conducted in R (RStudio Team, 2016; R Core Team, 2017) using the packages lme4 (Bates et al., 2015) and lmerTest (Kuznetsova et al., 2017).

### Typewriting Task

The experiment consisted of two tasks; a typewriting task and a mouse targeting task, alternating on a trial by trial basis. A trial of the typewriting task had a mean duration of 16.5 s (*SD* = 4.2) and the mean duration of a trial in the mouse targeting task was 6.6 s (*SD* = 0.7). In the present paper, we discuss the typewriting task, which is an adapted version of the task used by Hoeks et al. (2004).

**FIGURE 1:**
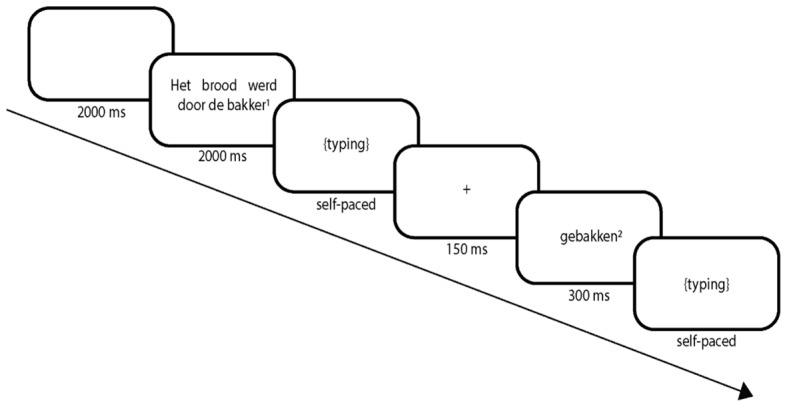
Schematic overview of a congruent trial of the typewriting task. (1) Het brood werd door de bakkers [the bread by the bakers]. (2) Congruent: gebakken [baked], incongruent: ‘bedreigd’ [threatened].

Each trial of the typewriting task started with a blank screen presented for 2,000 ms, followed by the first part of a sentence (i.e., all, but the last, word; **Figure 1**). After 2,000 ms the text was replaced with a blank screen, and the participants had to type the text they had read during sentence presentation. After finishing typing, the participants pressed *Enter* and a fixation cross appeared (1,500 ms). Then, the last word of the sentence appeared in the middle of the screen, which was replaced with a blank screen after 300 ms. The participant had to type the last word, again followed by pressing the Enter key to continue to the next trial. The sentences were presented in the Courier New font, with a size of 30 points.

Two types of sentences were presented, differing with regard to the semantic fit of the last word. The last word could either semantically fit with the first part of the sentence (congruent sentences; 75% of the sentences; e.g., het brood werd door de bakker gebakken. ‘the bread by the bakers baked.’ [the bread was baked by the baker]) or no semantic fit (incongruent sentences; 25% of the sentences; e.g., het brood werd door de bakker bedreigd. ‘the bread by the baker threatened.’[the bread was threatened by the baker]). Before the start of the experiment, sentence types were randomized with restriction, so that different incongruent sentences were separated by at least one congruent sentence. The duration of the experimental task was set to 120 min. As a consequence, the number of trials performed by each participant was dependent on how fast participants completed the trials (Young: *M* = 357, *SD* = 31.9; Middle-aged: *M* = 281, *SD* = 34.9).

### Subjective Measures

The Activation-Deactivation Adjective Check List (AD ACL; Thayer, 1989) was used to measure momentary activation states experienced by the participants. The participants indicated to what extent their mood could be described by 20 adjectives on a 4-point scale, ranging from 1 (definitely) to 4 (definitely not). The questionnaire categorizes the momentary state of activation into four clusters: general activation, deactivation/sleep, high activation and general deactivation.

### Performance Measures

The keylogging software registered a timestamp at the start and at the end of each keystroke with a code that referred to the identity of the pressed key (i.e., letter or backspace). Based on these markers, performance measures related to typing speed and typing accuracy were calculated for the sentence and the last word of each trial (**Table 1**). Typing speed is reflected in typing time, interkey interval, and stimulus–key interval. Typing time reflects the total time it takes to type the sentence or the last word of one trial. Interkey interval reflects the time between two subsequent keypresses, and is divided into four categories: general interkey interval (average time between two subsequent keypresses irrespective of the type of key), the letter–letter interval (average time between two subsequent letters), the letter–backspace interval (average time between a letter and a backspace), and the backspace–backspace interval (average time between two subsequent backspaces). The stimulus–key interval reflects the time between the offset of the last word of the sentence and the first keypress made to type this word. In addition, typing accuracy is reflected in the following measures: incorrect words, corrected words, backspace use, and backspace sequence length. The incorrect words represent the percentage of incorrectly typed words, the corrected words represent the percentage of words that are corrected, backspace use reflects the percentage of keystrokes that are backspaces, and backspace sequence length reflects the average key length of a sequence of consecutive backspaces.

**Table 1:** Keyboard-based performance measures related to typing speed and typing accuracy.

Typing speed
• Typing time
• Interkey interval
• General interkey interval:
• Letter–letter interval
• Letter–backspace interval
• Backspace–backspace interval
Typing accuracy
• Incorrect words
• Corrected words
• Backspace use
• Backspace sequence length


### EEG Recordings and Analysis

EEG activity was recorded using 20 Sn electrodes (F7, F3, Fz, F4, F8, FCz, C3, Cz, C4, CPz, P7, P3, Pz, P4, P8, PO7, PO8, O1, Oz, O2) attached to an electrode cap. Reference electrodes were placed behind each ear and a common electrode was placed on the collar bone. EOG activity was bipolarly recorded from two Sn electrodes placed at the outer canthi of both eyes for horizontal eye movements and above and below the right eye for vertical eye movements. Electrode impedance was kept below 5 kΩ.

We used independent component analysis (ICA) to identify eye blinks and eye movements (Jung et al., 2000). First, the data was down sampled to a rate of 500 Hz and filtered with a high pass filter of 0.5 Hz and a low-pass filter of 60 Hz. Independent component decomposition was conducted on epochs of 2,500 ms, starting 1,000 ms before sentence presentation. Epochs containing artifacts (amplitude < -120 or > 1,250 μV) were excluded from ICA decomposition. Channels that contained technical artifacts were replaced with interpolated values of surrounding electrodes (i.e., 0.8 channels on average per participant). In seven participants, resulting ICs did not reflect eye movements and eye blinks. IC decomposition in these participants was performed on 2,500 ms epochs around the last word of the sentence. Finally, ICs were copied to the original raw data, which was down-sampled to 500 Hz and band-pass filtered between 0.15 and 40 Hz. IC components that reflected eye blinks or eye movements (1 or 2 ICs per participant) were removed from the data.

Subsequently the data was segmented in epochs of 1,200 ms, starting 200 ms before the presentation of the last word of the sentence. Epochs in which the maximal absolute signal amplitude exceeded 110 μV were excluded from analysis. For four participants, settings were slightly adjusted because of larger average amplitudes in these datasets; in one dataset the high-pass filter was set on 0.5 Hz, in two datasets a threshold of 130 μV was used and in one dataset a threshold of 160 μV was used. The data was re-referenced to linked mastoids and aligned to a 200 ms pre-stimulus baseline.

The relationship between the independent variables (i.e., ToT, congruency, and age) and event-related brain activity (i.e., amplitude and latency of ERP components) was expressed in event-related regression coefficients (ERRCs) for every participant, channel, and time-point in the selected time-window. In other words, an ERRC expresses the relation between ERP amplitude and an independent variable such as ToT per participant (see Hauk et al., 2006; Miozzo et al., 2015 for a further discussion of ERRCs). From these modeled ERPs, the P1 was quantified as the mean amplitude in the 50 ms interval surrounding the most positive value between 100 and 160 ms after stimulus onset at the averaged signal of O1 and O2 (Boksem et al., 2005), and the N1 as the mean amplitude in the 50 ms interval surrounding most negative value between 160 and 210 ms post-stimulus at P7 and P8 (Boksem et al., 2005). The P3 was calculated as the mean amplitude surrounding the most positive value between 300 and 500 ms in the averaged signal of the electrodes CPz, Pz, P3, and P4 (Saliasi et al., 2013). Along with P3 amplitude, 50% fractional area latency was calculated in the P3 time-window (Hansen and Hillyard, 1980; Luck, 2005). The mean amplitude of the N400 component was computed on basis of the averaged signal of the electrodes Pz, Cpz, and Cz in the 250–500 post stimulus interval (Kutas and Federmeier, 2011).

### Procedure

Participants were instructed to abstain from alcohol 24 h before the experiment and from caffeine-containing substances for at least 12 h before the experiment. The experimental session started at 9 pm and took approximately 3.5 h. It was explained to the participants that the study was aimed at investigating differences in information processing between young and middle-aged employees. They were unaware that the analysis also included the factor ToT. Before the start of the task electrodes were applied, the participants were asked to hand in their phone and watch, and desk, chair, and screen support were adjusted according to the occupational health and safety guidelines. The participants filled out the AD-ACL and a questionnaire that contained general questions. Written task instructions were provided at the start of the experiment. For the typing task the participants were instructed to read and remember the first part of the sentence that appeared on the screen and type it as fast and accurate as possible after the text on the screen had disappeared. The same instruction applied to the last word of the sentence. Participants were asked to use the backspace key to correct for typing errors. To check whether the participants understood the task, they had to perform three practice trials. Thereafter, the participants performed the experimental tasks for 120 min, after which they filled out the AD-ACL again.

### Statistical Analysis

#### Subjective Data

For analyzing subjective fatigue, reflected in scores on the AD-ACL, we used mixed effects models with time of administration as a within-subject factor, and participant as a random factor. Missing values on the AD-ACL were replaced by the mean scores of the participant on the subscale of the missing value. Separately for both age groups and for each subscale of the AD-ACL, scores were centered around the mean of the first measurement. The raw data were used to calculate the means.

#### Performance Data

For the ToT analyses, trials were excluded from the analysis if scores on one of the behavioral measures in that trial was more than two standard deviations below or above the mean score, which was calculated within-subjects and across the whole experiment (*M* = 5.8%, *SD* = 2.9). Thereafter, trial data was pooled in 5-min bins, and average performance was computed for each bin. We used mixed effects models with age as a between-subject factor, ToT as a within-subject factor, and participant as a random factor (**Table 1**). Congruency (Cong) was included as an additional factor in the model used to analyze performance during typing of the last word. In the ToT analysis, a varying slope based on level of typing skill (i.e., the ability to type according to the 10-finger system without looking at the keyboard or not) was added to the model to correct for differences in typing skills across participants. The final models (i.e., factors included in the model) were based on model comparison using the likelihood ratio test. Based on these final models we calculated mean values at 20 min and at 120 min after the start of the experiment.

Data transformations were performed in order to realize that residuals of the models were normally distributed and did not show auto-correlation or heteroscedasticity. The dependent variable letter–letter interval was log-transformed. The dependent variables typing time, general interkey interval, stimulus–key interval, incorrect words, backspace sequence length, backspace use, and letter–backspace interval were root-transformed. The dependent variable corrected words was not transformed. To achieve linearity, the log-transform of the independent variable ToT was used in the models that included the number of backspaces and the backspace sequence length as dependent variables.

In addition to the previously described ToT analyses, we subjected the individual performance data to a linear regression with ToT as a fixed factor, which resulted in regression coefficients that described the effect of ToT on the dependent variables, separately for each participant. These performance-based regression coefficients were subjected to bivariate Pearson’s correlations to examine whether changes in the dependent variables were accompanied by changes in other dependent variables.

#### ERP Data

The mean ERRCs, reflecting the effect of ToT and congruency on the mean amplitude of the different components (i.e., P1, N1, P3, and N400), were subjected to the statistical analysis. *T*-tests were conducted to test whether the ERRCs significantly differed from zero, and ANOVAs were used to test whether the ERRCs significantly differed between age groups. To examine the relation between changes in event-related brain activity and changes in performance bivariate Pearson’s correlations were computed between the performance-based regressions coefficients and the mean ERRCs of the P3 amplitude and latency in the young and the middle-aged group. If the main analysis indicated a significant interaction (alpha < 0.05) between factors, follow-up analyses were performed, adjusting error rates according to Bonferroni.

## Results

### Subjective Measurements

Subjective fatigue increased during the experiment, as evidenced by a decrease in general activation scores from the start to the end of the experiment [*M*_before_ = 14.5, *M*_after_ = 11.1; χ^2^(1) = 40.09, *p* < 0.001], and an increase in scores on the deactivation/sleep scale [*M*_before_ = 10.1, *M*_after_ = 14.1; χ^2^(1) = 41.24, *p* < 0.001]. The level of tension, reflected in high-activation scores, did not change during the experiment [*M*_before_ = 6.5, *M*_after_ = 6.9; χ^2^(1) = 2.08, 0.122], although, participants felt less calm (i.e., decreased scores on the general deactivation scale) after the experiment compared to the beginning of the experiment [*M*_before_ = 17.2, *M*_after_ = 15.2; χ^2^(1) = 16.41, *p* < 0.001]. Scores on the AD ACL were not significantly influenced by age.

### Congruency

Congruency influenced task performance in both the young and the middle-aged group; in both groups stimulus–key interval was longer if the last word was incongruent with the first part of the sentence compared to endings that were congruent (young: *M*_cong_ = 250 ms, *M*_incong_ = 317 ms; middle-aged: *M*_cong_ = 431 ms, *M*_incong_ = 518 ms; **Table 2**). The effect of congruency was not dependent on ToT (**Table 2**), therefore the data was pooled over congruent and incongruent sentences for analyses concerning ToT.

**Table 2:** The effects of congruency, time-on-task, age, and their interaction on stimulus–key interval described by the chi-square (χ^2^) and the β-weights of the final model that describes the effects of congruency, time-on-task, age, and/or their interaction on stimulus–key interval.

Dependent variable	Cong χ^2^(1)	Age χ^2^(1)	ToT χ^2^(1)	Cong^∗^ Age χ^2^(1)	Cong^∗^ToT χ^2^(1)	Cong^∗^ToT^∗^ Age χ^2^(1)	β_Cong_ (*SE)*	β_Age_ (*SE)*
Stimulus–key interval	**14.83^∗∗∗^**	**140.48^∗∗∗^**	2.46	0.13	2.45	1.56	1.98 (0.16)	4.96 (1.19)


*The bolded values are the significant results. ^∗^*p* < 0.05; ^∗∗^*p* < 0.01; ^∗∗∗^*p* < 0.001.*

Early visual processing of the final word of a sentence, reflected in the P1 and N1 amplitude, was neither affected by congruency [P1, Cong: *t*(45) = 0.38, *p* = 0.707; N1, Cong: *t*(45) = -0.45, *p* = 0.656]. ERP components related to the further processing of stimulus information (i.e., P3 and N400), however, were affected by congruency; the P3 amplitude elicited by the incongruent last word was smaller compared to congruent words [P3, Cong: *t*(45) = -5.1, *p* < 0.001]. This difference was more pronounced in the young group than in the middle-aged group [P3, Cong^∗^Age: *F*(1,44) = 4.56, *p* = 0.038, young: *M* = 2.7, *SE* = 0.51, middle-aged: *M* = 1.1, *SE* = 0.52], however, this effect was not influenced by ToT [Cong^∗^ToT: *F*(1,44) = 1.86, *p* = 0.070]. Moreover, as expected, in sentences that contained a semantic violation, the last word of the sentence elicited a larger N400 amplitude as compared with congruent sentences [Cong: *t*(45) = -3.3, *p* = 0.002]. Processing of semantic violations, reflected in N400 amplitude, did not differ significantly between young and middle-aged participants [Cong^∗^Age: *F*(1,44) = 2.96, *p* = 0.093] and did not change with ToT [Cong^∗^ToT: *t*(45) = -1.6, *p* = 0.112]. β-weights, reflecting the N400 for each participant, and β-weights, reflecting the effect of congruency on stimulus–key interval for each participant, were negatively correlated in the young group [*r*(21) = -0.49, *p* = 0.017], indicating that the conflict processing was directly related to reaction speed in the young group. This relation did not reach significance in the middle-aged group [*r*(21) = 0.19, *p* = 0.374].

### Behavioral Measures

During the first 20 min of task performance, the average time to type the first part of the sentence remained stable in the young group (β_ToT_ = 0.06, *SE* = 0.10, *p* = 0.53), while the average typing time decreased in the middle-aged group (β_ToT^∗^Age_ = -0.15, *SE* = 0.075, *p* = 0.04), indicating that in this group the typing task was subject to practice effects. Therefore, data collected during the first 20 min of the task was excluded from further analysis.

#### Typing Speed

**FIGURE 2:**
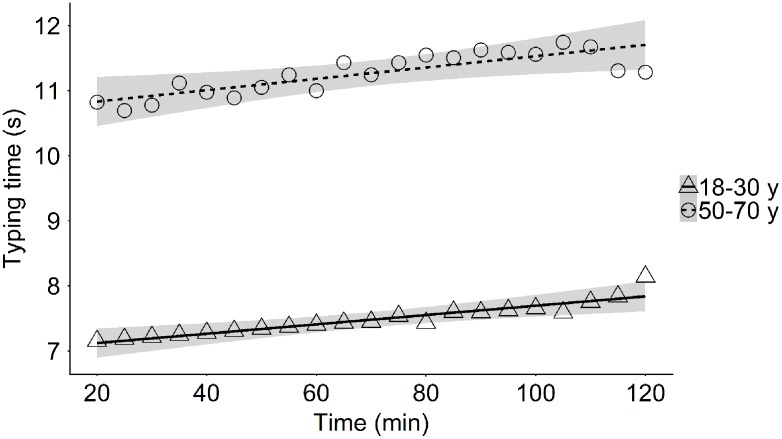
Mean typing time during sentence typing as a function of time-on-task in the young and the middle-aged group.

**FIGURE 3:**
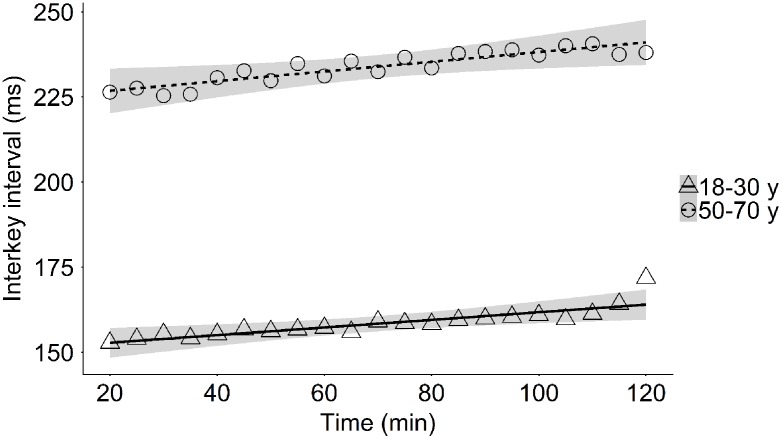
Mean general interkey interval during sentence typing as a function of time-on-task in the young and the middle-aged group.

In general, average typing speed was lower in middle-aged participants (typing time_sentence_: 11.3 s; typing time_lastword_: 3.45 s; general interkey interval_sentence_: 233 ms; general interkey interval_lastword_: 253 ms) than in young participants (typing time_sentence_: 7.36 s; typing time_lastword_: 2.08 s; general interkey interval_sentence_: 156 ms; general interkey interval_word_: 166 ms). In both groups the average typing time necessary to type the first part of the sentence increased with ToT (Young: *M*_t = 20 min_ = 7.25 s, *M*_t = 120 min_ = 8.03 s; Middle-aged: *M*_t = 20 min_ = 11.2 s, *M*_t = 120 min_ = 12.0 s; **Table 3** and **Figure 2**). This increase in typing time_sentence_ was, at least partly, due to an increase in the time between two subsequent key presses in the 20–120 min interval (general interkey interval_sentence_: Young_:_ *M*_t = 20 min_ = 152 ms, *M*_t = 120 min_ = 162 ms; Middle-aged, *M*_t = 20 min_ = 228 ms, *M*_t = 120 min_ = 240 ms; **Table 3** and **Figure 3**), as was evidenced by the relation between increased typing time_sentence_ during task performance and increased general interkey interval_sentence_ over the experiment in both young [*r*(21) = 0.88, *p* < 0.001] and middle-aged participants [*r*(21) = 0.60, *p* = 0.003].

**Table 3:** The effects of time-on-task, age, and their interaction on the different dependent variables of speed described by the chi-square (χ^2^) and the β-weights of the final models that describe the effects of time-on-task, age, and/or their interaction on the dependent variables.

Dependent variable	ToT χ^2^(1)	Age χ^2^(1)	ToT^∗^Age χ^2^(1)	β_ToT_ (*SE*)	β_Age_ (*SE*)	β_ToT^∗^Age_ (*SE*)
Typing time_sentence_	**9.40^∗∗^**	**38.40^∗∗∗^**	1.61	0.037 (0.004)	20.5 (2.67)	–
General interkey interval_sentence_	**8.64^∗∗^**	**43.65^∗∗∗^**	1.45	0.003 (0.0004)	2.79 (0.33)	–
Typing time_lastword_	0.42	**45.61^∗∗∗^**	**27.45^∗∗∗^**	-0.01 (0.005)	11.26 (1.62)	0.031 (0.006)
Stimulus–key interval_lastword_	**4.70^∗^**	**44.87^∗∗∗^**	**16.54^∗∗∗^**	0.0008 (0.0012)	4.17 (0.56)	0.007 (0.002)
General interkey interval_lastword_	**5.69^∗^**	**54.62^∗∗∗^**	**7.65^∗∗^**	-0.001 (0.002)	2.84 (0.32)	0.004 (0.001)


*The bolded values are the significant results. ^∗^*p* < 0.05; ^∗∗^*p* < 0.01; ^∗∗∗^*p* < 0.001.*

**FIGURE 4:**
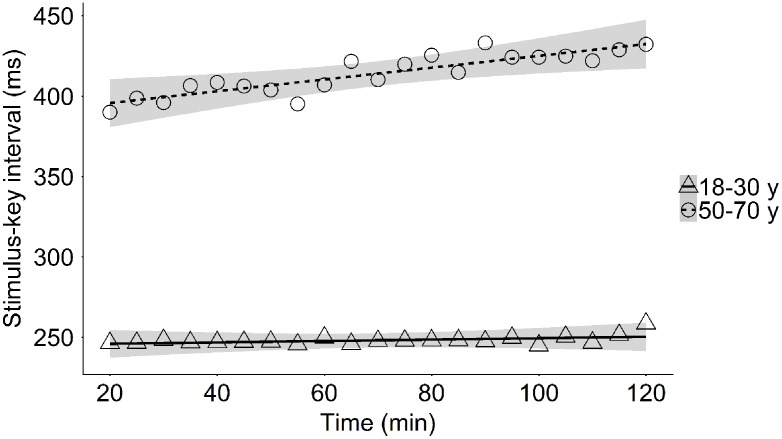
Mean stimulus–key interval as a function of time-on-task in the young and the middle-aged group.

In the young group, typing speed during the last word of the sentence did not significantly change with ToT (typing time: *M*_t = 20 min_ = 2.17 s, *M*_t = 120 min_ = 2.09 s; general interkey interval_lastword_: *M*_t = 20 min_ = 165 ms, *M*_t = 120 min_ = 167 ms; stimulus–key interval: *M*_t = 20 min_ = 244 ms, *M*_t = 120 min_ = 246 ms; **Table 3** and **Figure 4**). In the middle-aged group, however, typing speed during the last word of the sentence decreased during the 2-h experimental session (typing time_lastword_: *M*_t = 20 min_ = 3.43 s, *M*_t = 120 min_ = 3.62 s; general interkey interval_lastword_: *M*_t = 20 min_ = 245 ms, *M*_t = 120 min_ = 265 ms; stimulus–key interval_:_ *M*_t = 20 min_ = 397 ms, *M*_t = 120 min_ = 430 ms; **Table 3** and **Figure 4**). The middle-aged adults not only needed more time to type the last word of the sentence, they also initiated the first keypress later with increasing ToT.

#### Typing Accuracy

**FIGURE 5:**
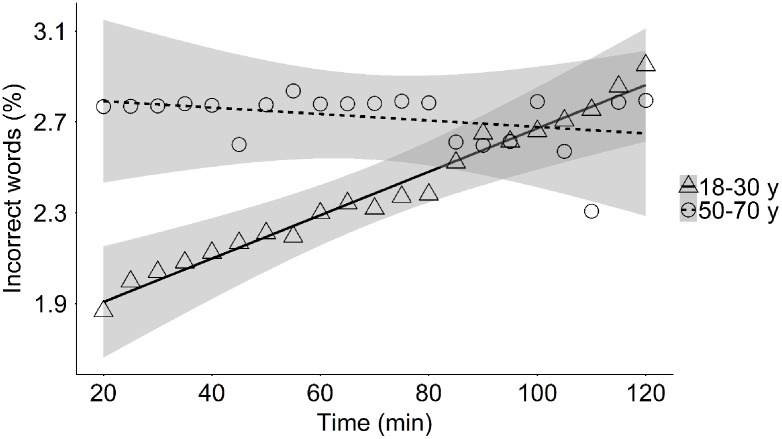
Mean percentage of incorrect words as a function of time-on-task in the young and the middle-aged group.

**FIGURE 6:**
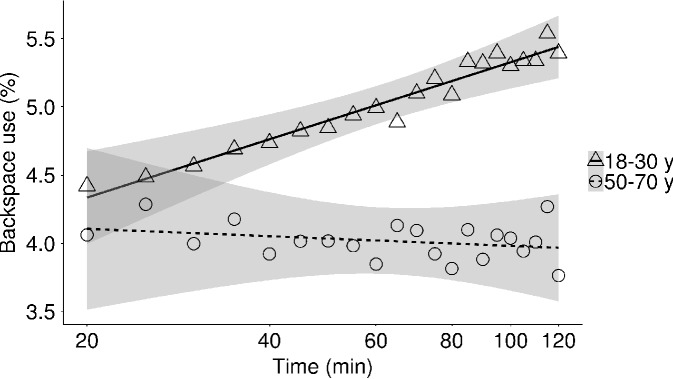
Mean backspace use as a function of time-on-task in the young and the middle-aged group.

In the young group the percentage of words that contained typing errors (incorrect words) increased from 1.8 to 2.7% over the experimental session, while this increase in typing errors was not observed in the middle-aged group (*M*_t = 20 min/120 min_ = 2.5%) (**Table 4** and **Figure 5**). Note, however, that early during the session the number of incorrect words was higher in the middle-aged group than in the young group. In addition, the average percentage of words that were corrected (corrected words) did not change significantly throughout the experiment in both the young and middle-aged group (*M*_t = 20 min/120 min_ = 13.1%; **Table 4**). Although the effect of ToT on the percentage of corrected words in the middle-aged group did not reach the level of significance, we observed that the percentage of error-corrections was positively related to the time to complete a sentence in this age group [*r*(21) = 0.53, *p* = 0.009], indicating that in the middle aged participants correcting errors takes time as reflected in increased typing time. In the young group, this relation was absent [*r*(21) = 0.28, *p* = 0.200]. While in the younger adults the percentage corrected words did not change over the experiment, the percentage of keystrokes that were backspaces did increase with increasing ToT (young: *M*_t = 20 min_ = 4.2%, *M*_t = 120 min_ = 5.4%; **Figure 6**). This increase in backspace use was due to an increase in backspace sequence length [i.e., backspace sequence length: *M*_t = 20 min_ = 1.9 keys, *M*_t = 120 min_ = 2.2 keys, **Table 5**; *r*(21) = 0.69, *p* < 0.001], indicating that young participants detected typing errors later with ToT. On average, backspace use did not change over the experiment in the middle-aged group (*M*_t = 20 min/120 min_ = 3.8%; **Table 4** and **Figure 6**). However, backspace sequence length decreased with prolonged task performance in this group (*M*_t = 20 min_ = 2.2 keys, *M*_t = 120 min_ = 1.9 keys, **Table 5**), indicating that middle-aged participants detected typing errors earlier with ToT.

**Table 4:** The effects of time-on-task, age, and their interaction on the different dependent variables of accuracy described by the chi-square (χ^2^) and the β-weights of the final models that describe the effects of time-on-task, age, and/or their interaction on the dependent variables.

Dependent variable	ToT χ^2^(1)	Age χ^2^(1)	ToT^∗^Age χ^2^(1)	β_ToT_ (*SE*)	β_Age_ (*SE*)	β_ToT^∗^Age_ (*SE*)
Incorrect words	**4.39^∗^**	2.62	**6.16^∗^**	0.002 (0.0007)	0.24 (0.15)	-0.002 (0.0010)
Corrected words	1.28	1.57	1.64	-	-	-
Backspace use	1.88	**3.90^∗^**	**15.29^∗∗∗^**	0.14 (0.036)	0.51 (0.27)	-0.20 (0.052)
Backspace sequence length	0.41	0.07	**7.29^∗∗^**	0.039 (0.027)	0.40 (0.17)	-0.10 (0.037)


*The bolded values are the significant results. ^∗^*p* < 0.05; ^∗∗^*p* < 0.01; ^∗∗∗^*p* < 0.001.*

**Table 5:** The effects of key sequence type (i.e., initiation of correction or during correction), age, and their interaction on the interkey interval type described by the chi-square (χ^2^) and the β-weights of the final models that describe the effects of time-on-task, age, and/or their interaction on the dependent variables.

Dependent variable	Type χ^2^(1)	Age χ^2^(1)	Type^∗^Age χ^2^(1)	β_Type_ (*SE*)	β_Age_ (*SE*)	β_Type^∗^Age_ (*SE*)
Letter–backspace interval	**219.8^∗∗∗^**	**54.27^∗∗∗^**	**15.52^∗∗∗^**	1.00 (0.025)	0.42 (0.054)	0.17 (0.038)
Backspace–backspace interval	0.11	**45.23^∗∗∗^**	**27.30^∗∗∗^**	0.087 (0.022)	0.45 (0.042)	-0.20 (0.032)


*The bolded values are the significant results. ^∗∗∗^*p* < 0.001.*

#### Key Sequence Effects

**FIGURE 7:**
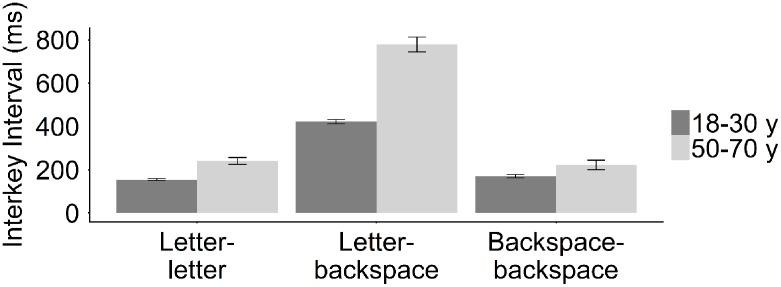
Mean letter–letter-(Young: *M* = 154 ms, *SE* = 4.38; Middle-aged: *M* = 240 ms, *SE* = 16.41), letter–backspace-(Young: *M* = 420 ms, *SE* = 9.55; Middle-aged: *M* = 784 ms, *SE* = 35.7), and backspace–backspace interval (Young: *M* = 222 ms, *SE* = 21.7; Middle-aged: *M* = 170 ms, *SE* = 7.03) in the young and in the middle-aged group.

The increase in typing time with increasing ToT was reflected in an increase in general interkey interval in both age groups. However, the results showed that average time between two subsequent key presses was dependent on the specific sequence of keys. More specifically, in both age groups the initiation of an error-correction (i.e., letter–backspace interval) resulted in longer interkey intervals as compared to the letter–letter intervals (**Table 5** and **Figure 7**). This effect was more pronounced in the middle-aged group than in the young group (**Table 5** and **Figure 7**). In addition, in the young group typing speed during error-corrections (i.e., backspace–backspace intervals) was slower than during correct typewriting (i.e., letter–letter intervals). In the middle-aged group we observed the opposite effect, that is, typing speed during error-corrections was faster than during correct typewriting (**Table 5** and **Figure 7**). These results showed that error-corrections resulted in slower typewriting in the younger adults, which suggests that the increase in general interkey interval in this group could, at least partly, be related to increased backspace use. This relation between backspace use and the interkey interval was further evidenced by the finding that the increase in backspace sequence length with ToT was correlated with an increase in general interkey interval in the young group (see accuracy). Since we established that typing speed during error-corrections is lower than typing speed during correct typewriting, these results suggest that the increase backspace sequence length in the young group result in decreased speed with ToT, reflected in increased general interkey intervals. Moreover, if we excluded typing speed during backspace use from the ToT analysis, the letter–letter interval during correct typewriting still decreased over the experiment in both age groups, however, this effect was now smaller in the young group (Young_:_ *M*_t = 20 min_ = 151 ms, *M*_t = 120 min_ = 154 ms; Middle-aged: *M*_t = 20 min_ = 232 ms, *M*_t = 120 min_ = 246 ms; **Table 6**), thereby providing additional evidence for the supposition that the increase in backspace sequence length with ToT is, at least partly, responsible for the increase in general interkey intervals in the young group.

**Table 6:** The effect of time-on-task, age, and their interaction on the different types of interkey interval described by the chi-square (χ^2^) and the β-weights of the final models that describe the effect of time-on-task, age, and their interaction on correct typewriting.

Dependent variable	ToT χ^2^(1)	Age χ^2^(1)	ToT^∗^ Age χ^2^(1)	β_ToT_ (*SE*)	β_Age_ (*SE*)	β_ToT^∗^Age_ (*SE*)
Letter–letter interval	**8.87^∗∗^**	**50.34^∗∗∗^**	**4.59^∗^**	0.0004 (0.00008)	0.43 (0.05)	0.0002 (0.0001)


*The bolded values are the significant results. ^∗^*p* < 0.05; ^∗∗^*p* < 0.01; ^∗∗∗^*p* < 0.001.*

### ERP Measures

**FIGURE 8:**
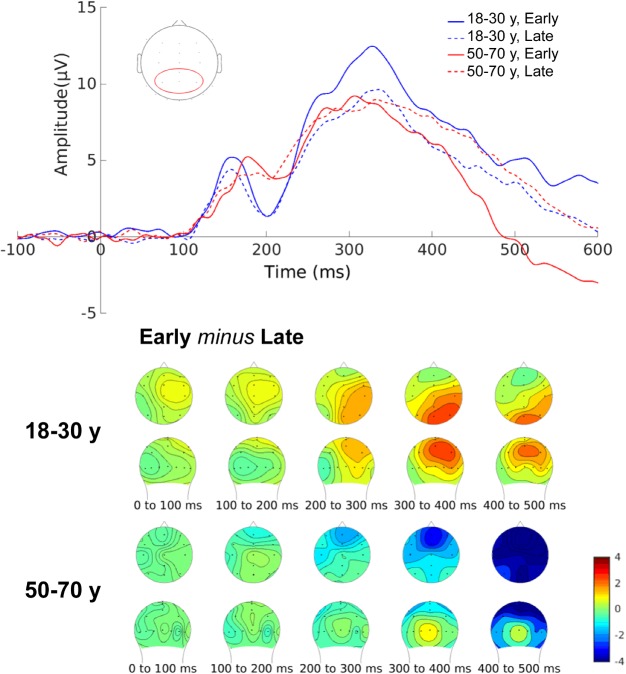
Effect of time-on-task on regression-derived ERP amplitude of the P3 component in young and middle-aged adults. Electrodes Cpz, Pz, P3, and P4 were used to model the ERP data. The modeled ERP early in the session describe the data 20 min after the start of the experiment. The modeled ERP late in the session describe the data 120 min after the start of the experiment.

Early visual processes, reflected in the P1 and the N1 ERP components, were not affected by ToT in neither the young nor the middle-aged group [P1, ToT: *t*(45) = 1.15, *p* = 0.256; ToT^∗^Age: *F*(1,44) = 0.21, *p* = 0.651; N1, ToT: *t*(45) = -0.71, *p* = 0.481; ToT^∗^Age: *F*(1,44) = 0.01, *p* = 0.922]. With regard to P3 amplitude, we observed a decrease in P3 amplitude with ToT in the young group [ToT^∗^Age: *F*(1,44) = 7.40, *p* = 0.009; Young, ToT: *t*(45) = -3.03, *p* = 0.006; **Figure 8**], which was less pronounced in the middle-aged group [middle-aged, ToT: *t*(45) = 1.50, *p* = 0.139]. Fractional area latency of the P3 increased with ToT in the middle-aged group [ToT^∗^Age: *F*(1,44) = 4.90, *p* = 0.032; middle-aged, ToT: *t*(45) = 2.78, *p* = 0.011], however, this effect was less pronounced in the young group [young, ToT: *t*(45) = 0.44, *p* = 0.662].

Deteriorations of performance in the young group (i.e., increased typing time, general interkey interval, and incorrect words) were accompanied by a decrease in the amplitude of the P3 [typing time: *r*(21) = -0.62, *p* = 0.002; general interkey interval: *r*(21) = -0.66, *p* < 0.001; incorrect words: *r*(21) = -0.52, *p* = 0.011]. An opposite trend was observed in middle-aged group, that is, in middle-aged participants an increase in the amplitude of the P3 was associated with an increase in general interkey interval [*r*(21) = 0.43, *p* = 0.043]. Decline in other keyboard-based performance measures did not correlate with changes in the P3 amplitude and/or latency in both the young and the middle-aged group.

## Discussion

In the present study, we investigated whether and how the effects of mental fatigue are modulated by age, and if we could validate the use of measures based on typing behavior as an index of the effects of mental fatigue on different aspects of cognition. We aimed to achieve this goal by examining the effects of mental fatigue, induced by prolonged typing performance, in young and middle-aged adults. In line with previous research, our results showed that participants experienced increased feelings of mental fatigue at the end of the experiment compared to the start of the experiment (e.g., Bonnefond et al., 2011; Lorist and Faber, 2011). In addition to the increase of subjectively reported fatigue, we observed that different measures based on typing behavior were influenced by 2 h of prolonged task performance, confirming that typing behavior could be used as an index of mental fatigue. In both the young and the middle-aged group a decrease in typing speed was reflected in larger general interkey intervals (i.e., the time between two subsequent keypresses). Although typewriting slowed down in both the young and the middle-aged group as a result of mental fatigue (i.e., increase in typing time and larger general interkey intervals), mental fatigue only influenced accuracy in the young group (i.e., increase in incorrect words and in backspace use in order to correct wrong keypresses). These findings are consistent with previous studies showing that older adults are reluctant to make mistakes and act more error-aversive than younger adults (Starns and Ratcliff, 2010), while younger adults more often adopt a strategy that emphasizes speed than older adults (Rabbitt, 1979; Lucci et al., 2013).

The first aim of the present study was to investigate the influence of age on the effects of mental fatigue. In general, the young participants were faster and performed more accurately than the middle-aged participants. Although typing skill has been found to be the largest predictor of differences in typewriting performance between age groups (Salthouse, 1984), the assessed level of skill of our participants (e.g., the ability to type according to the 10-finger system without looking at the keyboard) did not differ between age groups. Moreover, both groups worked a similar number of hours on a computer on a daily basis. Irrespective of these baseline differences, we observed a similar effect of mental fatigue on typing speed in both age groups. More specifically, the time that was needed to type the first part of a sentence increased over the experiment in both the young and the middle-aged groups, indicating that in both groups typing speed decreased as a result of mental fatigue. This effect was at least partly due to an increase in the time between two subsequent keypresses, suggesting that the general interkey interval could be used as an index of mental fatigue in both young and middle-adults.

After typing the first part of the sentence, the final word of the sentence was presented. This word could either be congruent or incongruent with the first part of the sentence. We found that the interval between the presentation of the last word and the first keystroke was longer for incongruent words than for congruent words in both age groups. Moreover, in accordance with results of the study of Hoeks et al. (2004), in both the young and in the middle-aged adults incongruent words were accompanied by a more negative deflection over central-posterior brain areas 400 ms after stimulus presentation as compared with congruent words. This enlarged N400 indicates that both groups processed semantically conflicting information differently than semantically correct sentences (Kutas and Van Petten, 1988). In addition to the effects of congruency, we found that the time necessary to type the final word of the sentence increased with increasing ToT. The observed effects of ToT on stimulus–key interval, general interkey interval and typing time were most pronounced in the middle aged participants. In both age groups, mental fatigue did not influence conflict processing either on a behavioral or neural level, making it unlikely that the congruency manipulation contributed to these differences in typing speed between the young and the middle-aged group during the final word of the sentence. In line with Hess and Ennis (2011), one might argue that these differences are related to the amount of effort that middle-aged and young participants invested in the task, that is, middle-aged participants might have invested greater effort to successfully perform the task than the younger adults. This enhanced cognitive engagement might have resulted in higher costs associated with task performance in the middle-aged group (Cappell et al., 2010; Ennis et al., 2014). As a result typing speed during the final word of the sentence could have been more prone to mental fatigue in this group compared to the young group.

Additional to the effects of mental fatigue and age on typing speed, we observed effects of prolonged task performance on typing accuracy. We found that with ToT the young participants showed an increase in words containing typing errors which was correlated with a decrease in P3 amplitude, an ERP component that has been associated with attention and memory operations (Polich, 2007). These results are in line with findings of Hopstaken et al. (2015), who established a strong link between task disengagement, as measured by a decrease in P3 amplitude, and mental fatigue. As a result of fatigue-related diminished cognitive engagement in the young adults, cognitive control processes requiring attention and memory seem to deteriorate, resulting in less efficient performance, as reflected in the increase in incorrectly typed words. Although participants were explicitly instructed to correct mistakes by using the backspace key, the number of error-corrections over the experiment remained stable, indicate that young participants did not compensate for reduced typing accuracy. Moreover, in the young group we observed an increase in the length of a backspace sequence with prolonged task performance, which indicates that young participants detected their mistakes later as they became fatigued. Despite the fact that middle-aged participants started out performing with lower accuracy than young participants, we did not find an additional decline in accuracy with increasing ToT in this group. More specifically, both the mistakes that were corrected and the mistakes that were not corrected by the backspace key remained stable over the experiment in the middle-aged group. In addition, we found a decrease in the backspace sequence length over the experiment in the middle-aged group, indicating that middle-aged participants detected their mistakes earlier with ToT. These results provide further support for the relation between error-aversive behavior and age (Hertzog et al., 1993; Starns and Ratcliff, 2010).

In the present experiment, backspace use indirectly affected typing speed, as it was positively correlated with the number of letters that had to be typed, and therefore, with the time that was needed to complete a sentence. Besides this obvious effect of backspace use on typing speed, we observed that young participants slowed down before error-corrections (i.e., letter–backspace interval between the last key press before a backspace and the first backspace) and during error-corrections (i.e., backspace–backspace interval between backspaces), which is in line with findings of Kalfaoğlu and Stafford (2014), who also found a decrease in typing speed before and during error-corrections. Both these effects of backspace use on typing speed are important for the interpretation of the effects of mental fatigue on typing speed observed in the present experiment. Firstly, the young group, and not the middle-aged group, used the backspace key more often with ToT because they detected their mistakes later, and subsequently had to delete and re-type more letters, resulting in increased time necessary to type the sentence. Secondly, we observed decreased typing speed with ToT in the young group as a result of the increase in backspace sequence length, because typing speed during error-corrections is lower than during correct typewriting^1^. These findings suggest that backspace use, besides general slowing, is responsible for the decrease in typing speed during the experiment in the young group, while general slowing, by itself, seems to explain the decrease in typing speed with increasing ToT in the middle-aged group.

In sum, our results revealed that changes in typewriting performance with increasing ToT seemed to have a different cause dependent on age. Altogether, the effects of ToT on different measures of typing performance confirm that middle-aged adults are reluctant to make mistakes and act more error-aversive than younger adults (Starns and Ratcliff, 2010), while younger adults more often adopt a strategy that emphasize speed than older adults (Rabbitt, 1979; Lucci et al., 2013), and that these differential strategies are visible in the ecologically sensitive typing task.

The second aim of our study was to investigate if we could validate the use of measures based on typing behavior as an index of the effects of mental fatigue on different aspects of cognition. Although the task challenged mental capabilities, typewriting also demands physical activity from the participant. Previous research showed that prolonged physical activity can result in physical fatigue. There are several reasons, however, why physical fatigue probably did not play a role in our experiment. Firstly, previous research showed that maximal voluntary contractions reach levels of only 6–20% during typewriting (Martin et al., 1996), which has been found to be too low to interfere with task performance (Lorist et al., 2002). Secondly, Kimura et al. (2007) established that muscle fatigue, measured by changes in the trapezius muscle, did not occur during 2 h of prolonged typing performance, except if 1 kg weight was added to each wrist. Lastly, the typewriting task alternated with a mouse targeting task during which participants did not use the keyboard, resulting in decreased physical load as compared with continuous typing. Combining previous literature with the characteristics of our design, we can conclude that physical fatigue most likely did not play a role in our experiment. Therefore, we would suggest that different measures based on typewriting performance can provide information about the influence of mental fatigue.

Brain activation, and specifically ERPs, can provide more insight in the cognitive processes underlying the described decline in typing performance as a result of mental fatigue. For example, the supposition that the general interkey interval could be used as an index of mental fatigue is supported by the relation between typing time, the general interkey interval, and brain activity in the young group. More specifically, we observed a decrease in the P3 amplitude over the experiment in the young group, which was related to an increase in typing time and longer general interkey interval. These effects were not related to a change in early visual processes (i.e., no change in P1 and N1 amplitude with ToT) in both age groups, indicating that sensory processes were not affected by mental fatigue, and suggesting that both groups were still sufficiently engaging in the task at the end of the experiment to be able to perceive visual information presented on the screen. Findings concerning the P3 amplitude were in line with Hopstaken et al. (2016), who observed a decrease in the P3 amplitude with ToT, which was not correlated with eye-movements away from task-relevant areas of the screen, suggesting that the decrease was related to lowered attention and memory processes involved in the further processing of task relevant stimuli. Besides a decrease in the P3 amplitude with ToT, Hopstaken et al. (2016) found an increase in the P3 amplitude if the benefits of finishing the task improved (i.e., increasing monetary rewards) at the end of the experiment, indicating that changes in the P3 amplitude are related to a cost-benefit evaluation of effort (Boksem and Tops, 2008; Hockey, 2011). They concluded that young adults are able to disengage from a costly fatiguing tasks to prevent excessive use of cognitive resources, and save these resources for more rewarding situations.

Although the P3 amplitude showed a similar trend over time in the young as in the middle-aged group, the decrease did not reach significance in the middle-aged group. In contrast, the fractional area latency increased in the middle-aged group, while this effect was less pronounced in the young group. These differential effects could indicate that cognitive processes were affected differently by mental fatigue in young and middle-aged group, that is, young adults might show more decline in the quality of processing, while processing speed declines more in middle-aged adults. Wascher and Getzmann (2014) also investigated the interaction between age and mental fatigue in age groups comparable to the groups we examined in the present experiment. In their study participants performed a visual spatial inhibition of return task. Wascher and Getzmann (2014) found similar results concerning the P3 amplitude: the P3 amplitude decreased over the experiment in the young group, however, not in the middle-aged group. In addition, they found that only middle-aged participants showed a decline in performance as a result of mental fatigue. Based on these findings they argued that adaptation mechanisms might differ between young and middle-aged adults. Behaviorally, our results showed a slightly different pattern, that is, young participants slowed down as a result of mental fatigue. However, in line with Wascher and Getzmann (2014), different adaptation mechanisms were at play: speed was affected more in the middle-aged group, whereas accuracy only declined in the young group.

Irrespective of the effects of mental fatigue, the results of the present study also showed a relation between typing performance and the N400 component, reflecting conflict processing. As expected, typing speed was affected by the processing of semantically conflicting information; participants slowed down when the text they typed was not fitting well with the context.

In sum, top-down cognitive control (e.g., attentional and working memory processes) is most susceptible to the effects of mental fatigue in both young and middle-aged adults. These results are in line with previous research that mental fatigue mainly has an effect on cognitive control processes (e.g., Lorist et al., 2005; Lorist and Faber, 2011), requiring the investment of effort (Dehaene et al., 1998), whereas bottom-up processes, requiring less effort, are relatively unaffected by mental fatigue. Although both age groups show a decline in cognitive control, young adults seem to demonstrate a larger decline in the quality of processing, whereas middle-aged adults show a larger decline in processing speed. These effects directly relate to the behavioral patterns found in the present study, which illustrate that middle-aged adults are reluctant to make mistakes and act more error-aversive than younger adults (Starns and Ratcliff, 2010), while younger adults more often adopt a strategy that emphasize speed than older adults.

## Author Contributions

MdJ, JJ, and ML conceived and designed the experiment. MdJ collected the data and analyzed the data. MdJ, JJ, AP, and ML wrote the manuscript.

## Conflict of Interest Statement

The authors declare that the research was conducted in the absence of any commercial or financial relationships that could be construed as a potential conflict of interest.

## Footnotes

1 The second result was supported by our finding that the effect of mental fatigue on the interkey interval decreased in the young group if we excluded error-corrections from the analysis.
